# Pallidal alpha activity is an electrophysiological biomarker of symptom severity in obsessive-compulsive disorder

**DOI:** 10.21203/rs.3.rs-9316385/v1

**Published:** 2026-04-20

**Authors:** Matteo Vissani, Ali Tafreshi, Clemens Neudorfer, Pranav Nanda, Alan Bush, Sahand Babapoor-Farrokhran, Andreas Horn, Joan Camprodon, Darin Dougherty, Cristina Cusin, Robert Richardson

**Affiliations:** Massachusetts General Hospital; Massachusetts General Hospital; Harvard Medical School; Massachusetts General Hospital; Massachusetts General Hospital; Massachusetts General Hospital; University Hospital Cologne; Massachusetts General Hospital Harvard University; Department of Psychiatry, Massachusetts General Hospital, Harvard Medical School, Boston, MA, USA; Department of Psychiatry, Harvard Medical School, Boston, MA, USA; Massachusetts General Hospital; Massachusetts General Hospital

## Abstract

Clinical efficacy of deep brain stimulation (DBS) for obsessive–compulsive disorder (OCD) remains limited by the absence of objective neural biomarkers to guide targeting, programming, and longitudinal optimization of therapy. Using sensing-enabled DBS devices, we analyzed intracranial local field potentials recorded longitudinally from thirteen patients with treatment-refractory OCD implanted in the ventral capsule/ventral striatum (VC/VS). We identify the periodic component of alpha-band activity (7–14 Hz) as a robust electrophysiological correlate of OCD symptom severity. At the initial clinical evaluation, periodic alpha power explained inter-individual variance in symptom severity, and within individuals it tracked longitudinal symptom fluctuations during chronic treatment. Spatial mapping localized the source of this periodic alpha activity to the anterior external globus pallidus (GPe), aligning with stimulation contacts selected as therapeutically optimal during clinical monopolar review. Together, these findings identify anterior GPe alpha oscillations as a state-dependent and spatially specific biomarker of OCD severity, with direct implications for electrophysiology-informed DBS programming and the development of adaptive neuromodulation strategies.

## Introduction

Obsessive–compulsive disorder (OCD) is a chronic and frequently disabling psychiatric illness, defined by intrusive, unwanted thoughts (obsessions) and repetitive, ritualistic behaviors (compulsions) performed to alleviate distress or prevent feared events^1^. Despite significant advances in pharmacological and behavioral therapies, up to 20–30% of individuals with OCD remain severely symptomatic and are classified as treatment–resistant^1-3^. For these patients, deep brain stimulation (DBS) has demonstrated substantial clinical benefit, with response rates of 50–70% across anatomical targets and an average symptom reduction of 40%^1-4^. Common targets for DBS in OCD include the ventral capsule/ventral striatum (VC/VS), nucleus accumbens (NAcc), bed nucleus of the stria terminalis (BNST), anterior limb of the internal capsule, and anteromedial subthalamic nucleus^5,6^. These anatomical sites serve as entry points to the cortico–striato– thalamo–cortical circuit, which is involved in a wide range of cognitive, motivational, and emotional processes^7^. Aberrant activity within this circuit is thought to play a central role in the pathophysiology of OCD ^8-10^.

Recently, connectomic studies^11-14^ have refined the neurobiological framework of OCD by identifying pathways whose engagement is associated with robust symptom improvement when stimulated with DBS. However, while connectivity-derived models characterize dysfunctional OCD circuits anatomically — i.e., suggest *where* to stimulate — they leave the nature of dysfunction unclear, as they are insufficient for developing mechanistic models or biomarkers of effective DBS. The absence of objective biomarkers that track OCD symptom severity during DBS remains a major barrier^15,16^ to efficient programming and optimization of DBS and to the development of adaptive neuromodulation strategies^17^.

Recent advancements in DBS device technology now enable intracranial neural activity to be recorded directly from implanted electrodes during routine clinical therapy^18^. These recordings provide access to local field potentials (LFPs), which capture population-level neural dynamics within the stimulated neural tissue as patients transition between symptomatic states and treatment conditions, creating an opportunity for biomarker discovery. In prior work^19^, we leveraged this capability in a single patient with severe, treatment-resistant OCD and showed that alpha-band activity recorded from VC/VS electrodes covaried with symptom severity across extended ambulatory recordings and was modulated by both stimulation and medication state. Consistent with the potential relevance of this frequency range, a subsequent study^20^ from another group demonstrated that reduced daily periodicity of the VC/VS activity within the same band predicted long-term clinical response in OCD patients. These findings suggested that alpha-band activity may index a pathological network state relevant to OCD symptom expression. However, whether this signal generalizes across patients, localizes to a consistent anatomical substrate, or provides clinically actionable information for DBS programming remained unknown.

Here, we evaluated whether alpha-band activity may serve as a suitable intracranial biomarker for DBS in OCD at the group level. Using in-clinic longitudinal LFP recordings acquired across thirteen patients with severe, treatment-resistant OCD, representing to our knowledge the largest cohort of chronic intracranial recordings in OCD studied to date, we systematically assessed whether alpha activity satisfies key requirements for clinical translation: whether it relates to OCD symptom severity across patients, tracks symptom fluctuations within individuals over time, exhibits consistent anatomical location, and informs the selection of therapeutically effective stimulation contacts. Using a comprehensive spectral analysis pipeline, we parameterized the periodic (putative oscillatory) component of the power spectrum, related it to OCD symptom severity, and mapped it anatomically along the DBS lead trajectory. To strengthen methodological rigor in a field where no consensus analytic framework exists, we adopted a multiverse analysis framework^21^, systematically evaluating the robustness of results across multiple analysis pipeline choices, similar to approaches recently applied to Parkinson’s disease biomarkers^22^. Our results identify the periodic component of alpha-band activity as a reproducible intracranial correlate of OCD symptom severity and demonstrate that electrophysiological signals recorded from DBS electrodes can provide a readout to inform DBS therapy in OCD.

## Results

### Clinical outcome heterogeneity motivates the quest for electrophysiological biomarkers

**Figure 1:**
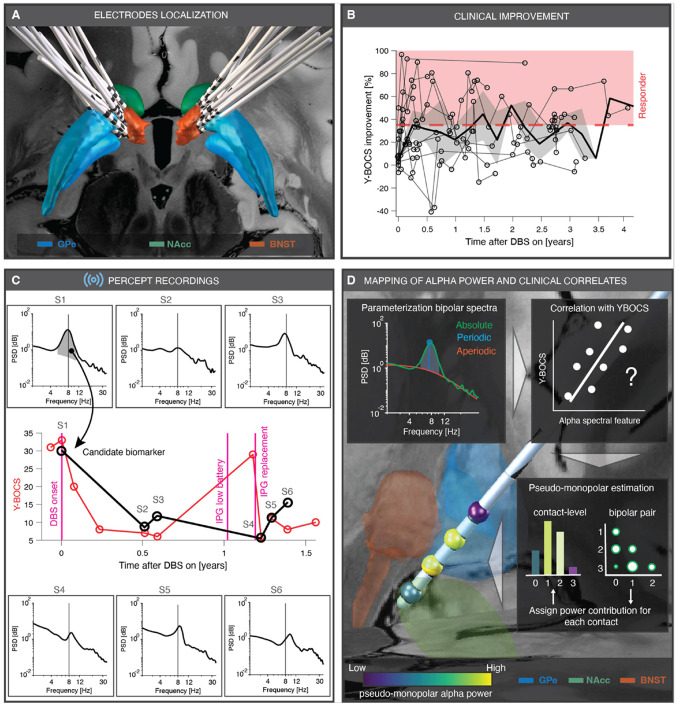
Sensing-enabled DBS devices enable the quest for electrophysiological biomarkers in OCD patients. **A,** Reconstruction of DBS lead placement in the ventral capsule/ventral striatum (VC/VS) in MNI space ( = 13). The external globus pallidus (GPe^23^, blue), nucleus accumbens (NAcc^23^, green), and bed nucleus of the stria terminalis (BNST^24^, orange) are visualized on a high-resolution post-mortem MRI reference. **B,** Evolution of OCD symptom severity after DBS, quantified using Yale–Brown Obsessive Compulsive Scale (Y-BOCS) scores obtained at postoperative clinic visits. Clinical improvement was expressed as percent change relative to preoperative baseline. Patients were deemed responders when achieving >35% Y-BOCS reduction (red patch above dashed line). Group-level improvement is shown as mean (thick black line) ± 95% confidence interval (gray shaded area), overlaid with individual patient trajectories (lines with circles). **C,** Example dataset from one patient (OP12), including Y-BOCS scores (red circles) collected across 11 clinic visits and six recording sessions (S1–S6) with bilateral intracranial signals acquired from the Medtronic Percept^™^ device. Power spectral density (PSD) from the left hemisphere shows a clear alpha peak (black vertical line) that, when plotted longitudinally (black circles), tracks fluctuations in Y-BOCS over time. Magenta vertical lines mark surgical events. **D,** PSD parameterization decomposed absolute power into periodic (cyan) and aperiodic (orange) components, with absolute power shown in green. Power was extracted either within the alpha band or within a ± 2.5-Hz window around the local alpha peak (cyan shaded area) and correlated with Y-BOCS scores from the first recording session S1, i.e., cross-sectional correlation analysis. These correlations were performed using different hemisphere-based strategies: pooling both hemispheres, isolating the left or right hemisphere, and summarizing within each hemisphere either by averaging or selecting the bipolar pairs with the maximum overall power. To visualize spatial distribution of alpha power, contact-level contributions were estimated from bipolar recordings (pseudo-monopolar estimates of power) and rendered as colored spheres (radius, 0.5 mm) in MNI space.

Thirteen patients with treatment-refractory OCD underwent bilateral implantation of a sensing-enabled DBS system (Medtronic Percept PC) (see **Supplementary Table 1** for clinical and demographic details). Electrode localization confirmed accurate placement of all DBS leads in the VC/VS, with trajectories traversing the external globus pallidus (GPe), bed nucleus of the stria terminalis (BNST), and nucleus accumbens (NAcc) (Fig. 1A). Patients were followed through a series of postoperative clinic visits aimed at monitoring clinical response and, if needed, optimizing stimulation parameters. Chronic stimulation produced clinically meaningful improvement overall (linear mixed-effect model (LME): main effect of time, *F* = 5.22, *P* = 2.4e-2), culminating in a 36.5±30.9% reduction at last follow-up (7 of 13 responders, 53.8%, *P* = 4e-3) (Fig. 1B). However, clinical outcomes were highly variable across patients (**Supplementary Fig. 1**), underscoring the clinical need of identifying electrophysiological biomarkers that can explain this heterogeneity and potentially inform DBS treatment. The Medtronic Percept PC system enabled the collection of time-domain neural recordings from all bipolar contact pairs (6 per hemisphere) during the DBS-OFF state using the BrainSense Survey mode, accessed via the Medtronic clinician programmer (Fig. 1C-D). The number of recording sessions per patient ranged from 1 to 6, enabling both cross-sectional and longitudinal analyses of neural activity in relation to OCD symptom severity. Electrode impedance remained stable (range: 1–6 kΩ; LME: main effect of time, *F* = 0.02, *P* = 8.97e-1; see **Supplementary Fig. 2**) over the duration of the DBS treatment. Additional demographic details, stimulation parameters, and sensing configurations are provided in **Supplementary Table 2**.

### VC/VS recordings exhibit prominent alpha and beta oscillations

We characterized the spectral properties of VC/VS activity obtained during the first recording session S1, which typically coincided with the first clinic visit and the programming and onset of DBS therapy (see Methods and **Supplementary Table 1** for exceptions). Visual inspection of absolute power spectra (no normalization) across all bipolar contact pairs displayed a prominent alpha peak and lack of a knee in the aperiodic component (**Extended Data** Fig. 1). To quantify this observation, we decomposed power spectra into periodic and aperiodic components applying a modified version^23^ of the *specparam*^24^ algorithm developed by our group. Spectral parameterization (mean ± s.d. goodness of fit, R = 0.98 ± 0.02; <2% of recordings with R < 0.9; **Supplementary Fig. 3** for details) identified alpha peaks in 75.7% of bipolar channel pairs and beta peaks in 88.2%. The distribution of alpha peak center frequencies exhibited a bimodal pattern with prominent modes around 8 Hz and 12 Hz, with most periodic alpha power confined within a ± 2.5 Hz window around the individual peak frequency. Beta peaks were also detected in 88.2% of recordings, although peaks in this range were less pronounced and less consistent in frequency compared to those in the alpha band. An aperiodic knee (knee frequency *f* < 1Hz) was identified in only 7.0% of spectra. Differences in the prevalence and spectral profiles of alpha and beta peaks between hemispheres are shown in **Supplementary Fig. 4**.

### Periodic alpha power explains OCD symptoms severity across patients

**Figure 2:**
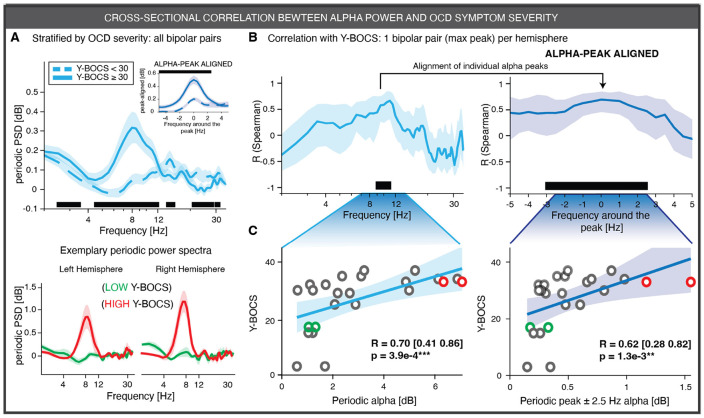
Periodic alpha power correlates with symptom severity in OCD patients. **A,** *(Top)* Bootstrapped average periodic power spectra (thick line; 10,000 iterations) and 95% confidence intervals (shaded area) stratified by symptom severity (Y-BOCS < 30, cyan dashed; Y-BOCS ≥ 30, cyan solid). Differences between groups were assessed using a cluster-based permutation test for unpaired data (10,000 permutations). Solid black line indicates significant clusters (α = 0.01). The inset shows the same analysis for periodic power spectral density (PSD) aligned to the individual alpha peak (−5 to +5 Hz window; blue). Only recordings acquired during the first recording session S1 were used (N = 144 bipolar channel pairs from 24 hemispheres in 13 patients). *(Bottom)* Representative periodic power spectra from the left and right hemispheres of two patients with low symptom severity (green) and high symptom severity (red). **B**, *(Left)* Bootstrapped average and 95% confidence interval of the frequency-wise Spearman correlation between periodic PSD and Y-BOCS scores. Significance was assessed using a cluster-based permutation test (α = 0.01; black line). *(Right)* Same analysis as in the left panel for periodic PSD aligned to the individual alpha peak (−5 Hz to +5 Hz window; blue). **C**, *(Left)* Correlation between periodic alpha power (7–14 Hz) and Y-BOCS scores across patients. Each circle denotes one patient. Spearman correlation coefficients and 95% confidence intervals are reported; the cyan shaded region shows the linear fit ±95% CI (visualization only). *(Right)* Same analysis as in the left panel using peak periodic power, defined as the average power within ±2.5 Hz around the individual alpha peak (blue). In **B–C**, only the bipolar channel pair with the highest power in each hemisphere was included (N = 24 hemispheres). Circles highlighted in red and green correspond to the patients shown in panel A. P values are summarized by asterisks (*P < 0.05, **P < 0.01, ***P < 0.001).

To evaluate whether VC/VS alpha activity could serve as an electrophysiological biomarker of OCD severity, we first tested a fundamental requirement: whether alpha power explains cross-sectional variance in symptom severity across patients using neural recordings obtained at the first recording session (S1). When patients were stratified by OCD symptom severity, those with more severe OCD symptoms showed increased low-frequency periodic activity (2–3 Hz; 4.5–12 Hz and 20.5–31 Hz; all P < 1e-4), with the strongest effect observed in the 4.5–12 Hz range, (Fig. 2A). When periodic alpha activity was aligned at the peak, we observed reduced alpha activity in less severe OCD patients. Frequency-by-frequency correlations between periodic power and Y-BOCS scores further demonstrated the spectral specificity of this association: only power within the alpha range showed a significant correlation with OCD severity (9–11.5 Hz, P < 1e-4, Fig. 2B) with a peak of R = 0.83 at 10.5 Hz. When alpha power was summarized at the hemisphere level, either by selecting the bipolar channel pair with the highest periodic alpha power or by averaging across all pairs, correlations with Y-BOCS scores remained significant (**Supplementary Fig. 5** and **Supplementary Table 3**). This was true for both the canonical alpha band (maximum bipolar pair: R = 0.70 [0.41–0.86], P = 3.9e-4 in Fig. 2C; mean across pairs: R = 0.46 [0.07–0.73], P = 2.59e-2) and for the ± 2.5 Hz narrowband centered on the individual alpha peak (maximum bipolar pair: R = 0.62 [0.28–0.82], P = 1.3e-3; mean across pairs: R = 0.51 [0.13–0.76], P = 1.25e-2 in Fig. 2C). These correlations were consistently stronger when only the left hemisphere was included in the analysis (**Supplementary Table 3)**. Taken together, these results indicate that periodic alpha power distinguishes between patients with higher and lower OCD severity and scales with symptom burden across individuals. Analyses based on absolute power spectra yielded qualitatively similar results, showing an elevation of broadband low-frequency activity (2–12 Hz) in patients with more severe OCD (**Supplementary Figs. 6 and 7**). However, this effect was accompanied by a steeper aperiodic slope (P = 9e-4), indicating that absolute power is a non-specific measure that conflates changes in periodic activity with changes in the aperiodic background of the power spectrum (**Supplementary Fig. 8**). In contrast, analyses based on relative (sum-normalized) spectra did not show a positive association in the alpha band but instead revealed a negative correlation in the beta range (13–35 Hz), reflecting the inter-frequency dependencies introduced by normalization (**Supplementary Fig. 9**). These results are therefore interpreted with caution and are described in detail in the **Supplementary Notes**.

### Periodic alpha power tracks longitudinal OCD symptom severity

**Figure 3:**
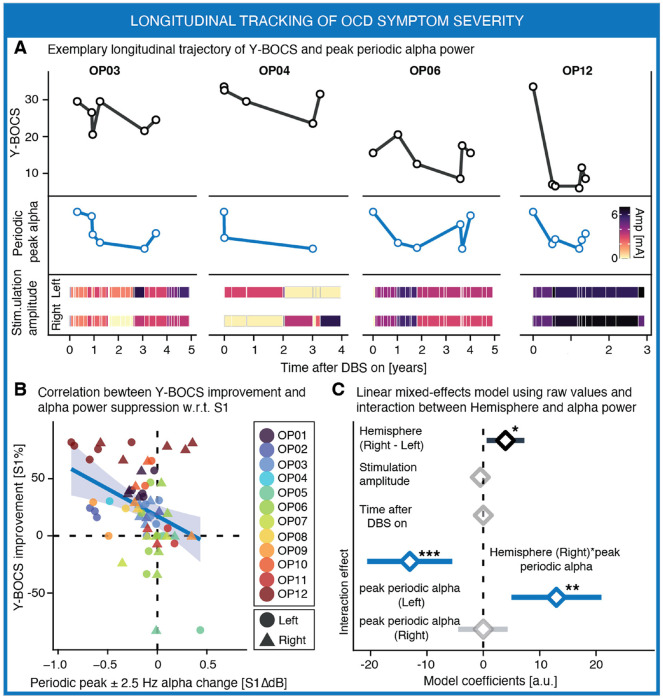
Periodic alpha power tracks longitudinal fluctuations in OCD symptoms severity during DBS treatment. **A,** Exemplary longitudinal trajectories from four representative patients illustrating Yale–Brown Obsessive Compulsive Scale (Y-BOCS) scores (*Top*, black), peak periodic alpha power in the left hemisphere (*Middle*, blue), and stimulation amplitude (*Bottom*) across recording sessions after DBS onset. Peak periodic alpha power was quantified within a ±2.5 Hz window centered on the individual alpha peak and normalized (min-max) in each DBS lead. Heat maps indicate stimulation amplitude (mA) delivered in the left and right hemispheres over time. **B**, Relationship between longitudinal changes in peak periodic alpha power and clinical improvement relative to the first recording session (S1). Each point represents one recording session. Circles denote the left hemisphere and triangles denote the right hemisphere; colors identify individual patients. Negative changes (as log-ratio) in alpha power reflect suppression of periodic alpha activity, whereas positive values of Y-BOCS improvement (as percentage) indicate clinical benefit. The solid line represents the Spearman regression with 95% confidence interval. **C**, Linear mixed-effects model relating Y-BOCS scores to periodic peak alpha power while accounting for stimulation amplitude and time since DBS onset. Model coefficients (diamonds) and 95% confidence intervals (obtained from the standard error (SE) reported in **Supplementary Table 5**) are shown. A significant interaction between hemisphere and peak periodic alpha power indicates that the relationship between alpha activity and symptom severity differs across hemispheres, with a stronger association in the left hemisphere. In **B-C**, only the bipolar channel pair with the highest power in each hemisphere was included. P values are summarized by asterisks (*P < 0.05, **P < 0.01, ***P < 0.001).

We previously showed that periodic alpha power correlated with Y-BOCS scores across patients at the first recording session (S1). However, a clinically useful electrophysiological biomarker for deep brain stimulation (DBS) should also track symptom fluctuations within individuals over time. We therefore examined whether longitudinal changes in periodic alpha activity accompany changes in OCD symptom severity. To test this, we analyzed all available recordings across patients (1–6 sessions per patient; N = 582 recordings from 12 patients) and quantified whether session-to-session changes in alpha power tracked changes in symptom severity relative to S1 (Fig. 3A). Positive changes in Y-BOCS reflected clinical improvement, whereas reductions in alpha power reflected suppression of oscillatory activity.

Across sessions, decreases in periodic alpha power were significantly associated with improvements in OCD symptoms (**Supplementary Table 4**). This relationship was observed when alpha activity was quantified in the canonical alpha band (maximum bipolar pair: R = −0.34 [−0.57 – −0.07], P = 6.05e-3; mean across pairs: R = −0.15 [–0.42–0.14], P = 2.53e-1) and was stronger when alpha power was measured within a narrowband window centered on the individual alpha peak (maximum bipolar pair: R = −0.37 [−0.61 – −0.09], P = 2.9e-4 also shown in Fig. 3B; mean across pairs: R = −0.29 [−0.55 – −0.01], P = 1.77e-2).

We next modeled the relationship between alpha power and Y-BOCS using linear mixed-effects (LME) models that accounted for repeated measurements within patients while including stimulation amplitude and time since DBS onset as covariates. In the primary model, hemispheres were pooled and an interaction term between hemisphere and alpha power was included (Fig. 3C). Peak periodic alpha power showed a significant association with symptom severity (maximum bipolar pair: β = 12.97 ± 3.97 (Mean ± 95th CI), F = 6.24, P = 1.47e-2; mean across pairs: β = 18.68 ± 5.73, F = 6.65, P = 1.19e-2). The hemisphere × alpha power interaction was also significant (maximum bipolar pair: F = 11.87, P = 9.72e-4, mean across pairs: F = 12.11, P = 8.67e-4), indicating that the strength of this association differed across hemispheres. Consistent with this interaction, hemisphere-specific models revealed a significant association between periodic alpha power and OCD symptom severity in the left hemisphere (maximum bipolar pair: β = 16.48 ± 5.02, F = 10.76, P = 2.29e-3; mean across pairs: β = 23.57 ± 7.23, F = 10.62, P = 2.43e-3), whereas no association was observed in the right hemisphere (all P > 0.4). LME results were qualitative similar when computing periodic alpha power in the canonical alpha frequency band (**Supplementary Table 5**).

As a complementary analysis, we compared periodic power spectra between the first recording session (S1) and the session corresponding to the greatest DBS-induced clinical improvement (ΔY-BOCS: 9.8 [5.2 14.8]; time between sessions: 408.8 [186 666] days), where the encoding of therapeutic electrophysiological biomarker would be expected to be maximal (**Extended Data** Fig. 2). Periodic spectra during the best-clinical session showed a reduction in low-frequency periodic activity (5–6.5 Hz and 8.5–14 Hz; all P < 1e-4 Fig. 5D), accompanied by a reciprocal increase in beta-range periodic power (18–28 Hz; P < 1e-4). This effect was driven by the left hemisphere (see **Supplementary Notes** and **Supplementary Fig. 10**), where electrode contacts were positioned more posteriorly (ΔY = 0.6 ± 0.5 mm, *P* = 1e-4) and laterally (ΔX = 1.2 ± 0.6 mm, *P* = 1e-4) than in the right hemisphere, resulting in contacts located closer to the GPe border (Δ distance = 1.47 ± 1.0 mm, *P* = 2.58e-2) and farther from the BNST border (Δ distance = 2.10 ± 0.9 mm, *P* = 2.7e-2).

Taken together, these results show that reductions in periodic alpha activity track longitudinal improvements in OCD symptom severity. These findings identify periodic alpha activity as a dynamic neural signal linked to the clinical state of OCD and support its potential as a candidate control signal for future closed-loop DBS therapies. Analyses based on absolute (**Supplementary Fig. 11**) and relative (**Supplementary Fig. 12**) spectra are reported in details in **Supplementary Notes** and **Supplementary Table 5**.

### Optimal contact prediction

**Figure 4:**
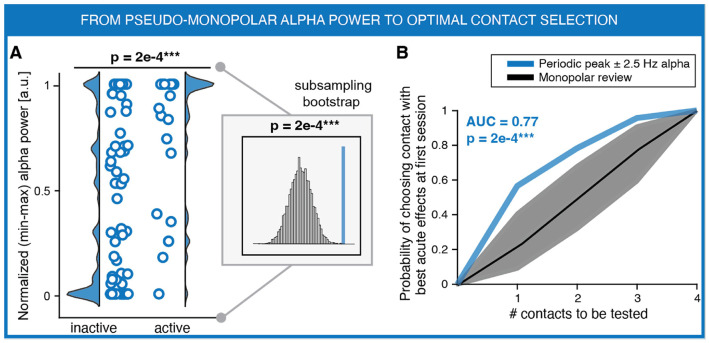
Periodic alpha power identifies optimal stimulation contacts. **A,** Distribution of peak periodic alpha power (blue) in inactive versus active contacts, defined as those chosen for stimulation during the first programming session (largely overlapping with the first recording session, S1). Peak periodic alpha power was quantified within a ±2.5 Hz window centered on the individual alpha peak. Each circle represents a contact. Inset shows comparison of alpha power in active contacts (vertical line) against a sample-balanced distribution of inactive contacts generated by subsampling (10,000 iterations) (N = 96 contacts across 24 hemispheres from 13 patients). **B,** Cumulative probability of selecting the clinically optimal stimulation contact based on the highest alpha power rank across contacts within each hemisphere. Dashed line and gray shaded area indicate prediction accuracy of the monopolar contact review with 95% confidence interval. Prediction capability was quantified as area under the curve (AUC; chance = 0.5, perfect prediction = 1) and tested against the manual mapping distribution. P values are summarized by asterisks (*P < 0.05, **P < 0.01, ***P < 0.001).

Programming DBS therapy for OCD relies on monopolar reviews in which individual contacts are sequentially tested to identify stimulation sites likely to provide therapeutic benefit. In psychiatric DBS targets such as the VC/VS, clinicians often rely on transient affective responses, most notably mirth or spontaneous smiling, as signs of target engagement. However, improvements in OCD symptoms typically emerge only weeks to months after stimulation adjustments, making it difficult to directly evaluate therapeutic efficacy during programming. Consequently, contacts are selected based on acute behavioral responses and clinical judgment, and their long-term therapeutic value becomes apparent only after extended follow-up. An electrophysiological signal linked to the OCD clinical state could therefore provide an objective marker to guide the selection of stimulation contacts. We therefore examined whether alpha activity predicts the contacts selected for therapeutic stimulation. Specifically, we tested whether alpha power could explain DBS programming decisions by predicting the optimal stimulating contact identified during the monopolar review at the first clinical visit, which coincided with the first recording session (S1) (Fig. 4). Contacts were classified as active or inactive depending on whether they were selected for therapeutic stimulation following the monopolar review. Active contacts therefore correspond to those that the clinical team deemed most promising for therapeutic response based on intra-clinical testing and acute behavioral effects.

To enable this comparison at the level of individual contacts, we applied a recently described approach that projects spectral features extracted from bipolar channel pairs onto pseudo-monopolar estimates that can be assigned to individual electrode contacts (see Methods; Fig. 2D and **Supplementary Fig. 13**). This framework allowed us to compare electrophysiological activity across contacts at the first recording session.

Periodic alpha power was significantly higher at contacts selected for stimulation than at inactive contacts both when periodic power was averaged in the narrowband window centered on the individual alpha peak (Fig. 4A; P = 2e-4) and in the canonical alpha band (**Extended Data** Fig. 3; P = 1e-3). These results were robust to potential imbalances in the number of active and inactive contacts, as a sample-balanced bootstrapping procedure yielded comparable results across all alpha metrics (inset plots in Fig. 4A and **Extended Data** Fig. 3A).

We next assessed whether alpha activity could predict the ranking of contacts for therapeutic stimulation. For each hemisphere, contacts were ranked according to alpha power and compared with the clinically selected optimal contact. Performance was quantified as the area under the curve (AUC) of the cumulative probability of selecting the optimal stimulating contact. Periodic alpha power significantly outperformed conventional manual review in identifying the optimal stimulation contact (**Extended Data** Fig. 3B; AUC = 0.73, P = 2.3e-3. When selecting only the top-ranked contact based on periodic alpha power, the probability of identifying the clinically optimal contact was 56.5%, increasing to 69.6% when the two highest-ranked contacts were considered. Predictive performance further improved when alpha activity was quantified at the individual alpha peak (Fig. 4B). Peak periodic alpha power achieved an AUC of 0.77 (P = 2e-4), with a probability of correctly identifying the optimal stimulating contact of 56.5% using the top-ranked contact and 78.3% when considering the two highest-ranked contacts.

To directly quantify the agreement between electrophysiology-based and clinically selected contacts, we measured the normalized rank distance between the clinically optimal contact (rank = 1 by definition) and the corresponding electrophysiological rank. Both canonical periodic alpha power (rank difference = 0.83 [0.44–1.26], P = 3.6e-3) and peak-aligned periodic alpha power (rank difference = 0.70 [0.35–1.09], P = 3e-4 showed significantly better alignment with clinical contact selection than conventional manual mapping and outperformed absolute alpha power (rank difference = 1.39 [0.87–1.89], all P < 3.2e-2), which showed no predictive capability (**Supplementary Fig. 14**). Relative alpha power showed comparable predictive performance to periodic alpha power (**Supplementary Fig. 15**) and is reported in the **Supplementary Notes**.

Together, these findings indicate that periodic alpha activity identifies stimulation sites associated with therapeutic benefit and predicts the contacts selected during clinical DBS programming. These results suggest that alpha activity within the VC/VS circuit may provide an objective electrophysiological marker to guide DBS contact selection.

### Anatomical mapping of alpha activity

The association between alpha power and therapeutically effective stimulation contacts suggested that this electrophysiological signal may arise from specific anatomical substrates within the VC/VS circuitry. We therefore examined the spatial distribution of alpha activity across recording sites (**Supplementary Table 6**).

**Figure 5:**
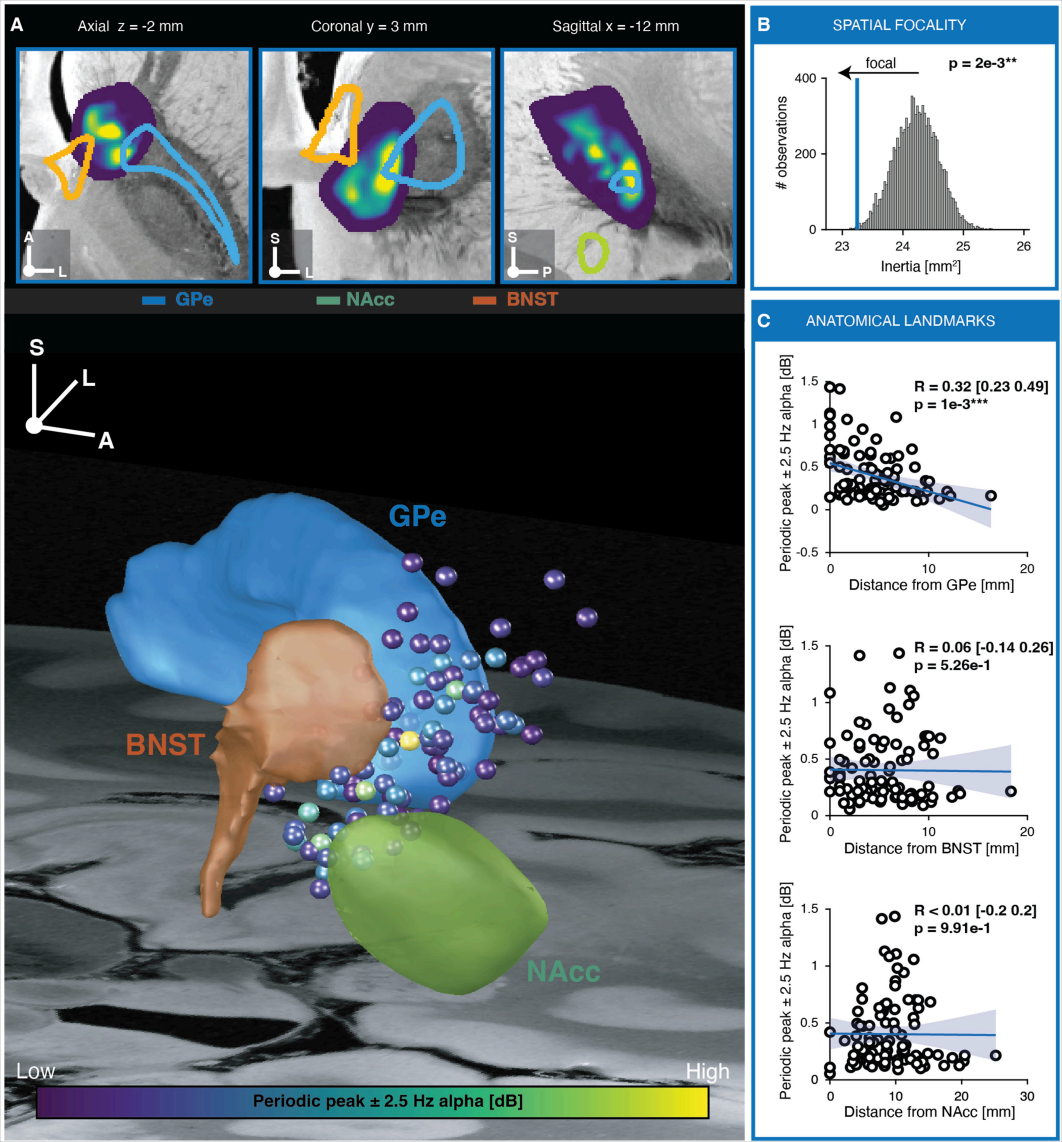
Periodic alpha power is localized in the anterior region of GPe. **A,** Two-dimensional heatmaps of pseudo-monopolar peak periodic alpha power shown in axial, coronal, and sagittal views, overlaid with subcortical parcellations from the Lead-DBS toolbox: external globus pallidus (GPe, blue) and nucleus accumbens (NAcc, green) from the OCD Response Tract Atlas^23^, and bed nucleus of the stria terminalis (BNST, orange) from the Human Hypothalamus Atlas^24^. Images are visualized against a high-resolution post-mortem MRI backdrop^25^. Three-dimensional visualization of pseudo-monopolar peak periodic alpha power without interpolation or smoothing (N = 96 contacts across 24 hemispheres from 13 patients) using colored spheres (radius, 0.5 mm). Peak periodic alpha power was quantified within a ±2.5 Hz window centered on the individual alpha peak. **B,** Inertia, an index of spatial dispersion of peak periodic alpha power (lower values indicate more focal clustering around a center of mass), for the observed distribution (vertical blue line) compared with a null distribution generated by randomly permuting contact locations (10,000 permutations). **C,** Correlation between pseudo-monopolar peak periodic alpha power and distance from each contact to the nearest border of the GPe, NAcc, and BNST. Each circle denotes one contact; Spearman correlation coefficients (sign inverted) and 95% confidence intervals are reported. Shaded region shows linear fit ±95% CI (for visualization only). P values are summarized by asterisks (*P < 0.05, **P < 0.01, ***P < 0.001).

To determine whether alpha activity at the first recording session (S1) localized to specific subregions of the VC/VS, pseudo-monopolar estimates of alpha power were assigned to the MNI coordinates of DBS lead contacts and projected into MNI space. Contact locations were distributed along the anterior limb of the internal capsule (ALIC) and surrounding gray matter structures including the nucleus accumbens (NAcc), bed nucleus of the stria terminalis (BNST), and external globus pallidus (GPe) (Fig. 5). The spatial centroid of recording sites exhibiting the top 5% of peak periodic alpha power was located in the anterior portion of the GPe (MNI coordinates: X = −10.12, Y = 4.07, Z = −4 mm). Visualization of raw 3D scatter points and corresponding 2D heatmaps in axial, coronal, and sagittal planes yielded the same localization without applying an a priori percentile threshold (Fig. 5A).

We next quantified the spatial focality of peak periodic alpha activity using two complementary approaches. First, periodic peak alpha power showed a significant correlation with the Euclidean distance from each contact to the spatial maximum (R = 0.27 [0.08–0.45], P = 7e-3) indicating that alpha power decreased with increasing distance from the peak location. Second, we calculated the moment of inertia of the spatial power distribution to estimate the radius of gyration (√I), representing the effective radius of an equivalent sphere with the same moment of inertia (Fig. 5B). Peak periodic alpha activity exhibited a significantly more focal spatial distribution than expected from a uniform distribution (I = 23.5 mm², √I = 4.82 mm, P = 2e-3). The convergence of these independent measures confirmed that the spatial distribution of periodic alpha activity was tightly associated with DBS lead position. Results were comparable when considering periodic alpha power averaged within the canonical alpha band.

Finally, we examined the relationship between alpha activity and the anatomical borders of structures within the VC/VS region. A significant correlation was observed only for the distance to the GPe border (R = 0.32 [0.23–0.49], P = 1e-3), whereas no consistent associations were detected for the NAcc or BNST (Fig. 5C). Results using absolute (**Supplementary Fig. 16**) and relative (**Supplementary Fig. 17**) alpha power are discussed in detail in **Supplementary Notes** and **Supplementary Table 6**.

Together, these findings indicate that alpha activity associated with OCD symptom severity reflects a spatially localized signal within the VC/VS region, peaking in the anterior GPe rather than arising from nonspecific volume conduction.

## Discussion

Electrophysiological biomarkers of OCD symptoms severity are critically needed for improving the efficacy of DBS treatment for treatment-refractory OCD. As an initial step toward establishing an objective biomarker, we previously reported in a single-patient case study^19^ that VC/VS recordings exhibited a prominent peak in the alpha-band that tracked fluctuations in OCD symptomatic state across thousands of hours of chronic sensing. Here, we extend this biomarker discovery effort to thirteen patients with severe, treatment-refractory OCD (the largest cohort studied with chronic, sensing-enabled DBS in OCD using longitudinal LFP recordings) who were implanted with clinically indicated, sensing-enabled DBS leads targeting the VC/VS. Using months-to-years of longitudinal, inclinic, high-resolution time-domain local field potential (LFP) recordings, we evaluated neural–symptom relationships across a broad analytic “multiverse” spanning spectral normalization and parameterization (absolute, periodic and relative power), frequency definition (frequency-wise analyses, canonical bands, and narrowband peak alpha), hemisphere sampling (pooled, left-only, right-only) and channel selection (mean across channels versus the channel exhibiting maximal alpha power). We adopted this multiverse framework^21^ — similar to that applied in the context of Parkinson’s disease biomarkers^22^ — to strengthen methodological rigor in a field where no consensus analytic standard has been established. Across our analyses, a consistent pattern emerged linking the periodic component of alpha-band activity in the VC/VS circuitry to the clinical state of OCD. First, periodic alpha power recorded at the initial programming session correlated with OCD symptom severity across patients. Second, periodic alpha activity tracked symptom fluctuations longitudinally within individuals across repeated clinic visits. Third, using pseudo-monopolar estimates derived from bipolar recordings, periodic alpha power predicted the stimulation contacts ultimately selected during clinical programming. Fourth, spatial mapping of pseudo-monopolar alpha estimates localized peak periodic alpha activity to the anterior external globus pallidus (GPe), where alpha power declined with increasing distance from the GPe border. Notably, associations between alpha activity and clinical symptoms were consistently stronger in the left hemisphere. Analyses based on absolute or relative power spectra yielded qualitatively similar but less consistent results, indicating that isolating the periodic component of the signal provides a more robust measure of disease-relevant neural dynamics. Together, these findings identify excessive periodic alpha activity localized to the anterior GPe as an electrophysiological correlate of OCD symptom severity and suggest that this signal may provide clinically actionable information for DBS programming and the development of adaptive neuromodulation strategies.

Our study advances the field in three critical key areas that are necessary to assess whether a candidate biomarker holds translational potential. First, we integrated spectral parameterization to address a fundamental limitation of conventional power-based analyses that conflate periodic and aperiodic components and introduce interdependencies across frequency bands^24-26^. Such factors complicate electrophysiological interpretation of findings and can obscure relationships with clinical variables^22^. Second, we implemented pseudo-monopolar mapping^27^ to estimate the contribution of power at each reconstructed contact using the Lead-DBS toolbox. Because bipolar recordings do not resolve individual contacts, pseudo-monopolar mapping is a necessary, if approximate, step toward clinical operationalization (see Pseudo-monopolar alpha power in Methods). Our results demonstrate that LFP biomarkers in OCD can be translated into actionable information for DBS programming. Third, we explicitly evaluated whether candidate biomarkers tracked symptom fluctuations within individuals over time. This property cannot be inferred from cross-sectional associations alone^22^ and is essential for biomarkers intended to guide adaptive DBS protocols.

Our findings are consistent with models positing that OCD arises from aberrant neural dynamics within cortico-striato-thalamo-cortical circuits^6,11,28,29^. Excess alpha activity may reflect pathological rhythmic coordination within these networks. Early intracranial studies^30-35^ suggested low-frequency neural activity within VC/VS-adjacent regions as physiological correlates of cognitive and affective processes relevant to OCD, including intrusive thoughts^31^, error monitoring^32^, reward processing^36^, and compulsive behavior^37^. In our study we observed a broad increase of low-frequency activity (< 11 Hz) in most severe patients that correlated with OCD symptom severity using absolute and relative power spectra. This is generally congruent with prior work using similar spectral normalization strategies (either absolute or relative) that reported changes in the delta band (1–4 Hz) in OCD patients in the cortico-striato-thalamo-cortical circuit during naturalistic provocation tasks^38-40^. Using LFPs from externalized leads implanted in BNST in a small cohort of OCD patients, acute VC/VS stimulation was associated with suppression of theta power and increases in higher frequency bands^35^. A recent acute post-operative symptom provocation study^39^ in a cohort of 11 OCD patients showed that delta and alpha power increase during compulsions across multiple basal ganglia structures, including GPe, NAcc, and anterior limb of the internal capsule. Interestingly, neither delta nor alpha power in GPe correlated with OCD symptom severity. Notably, most of these prior studies do not report clear oscillatory peaks in the low-frequency range. In this context, our findings suggest that at least some previously reported broadband low-frequency effects may reflect shifts in the aperiodic component of the power spectrum rather than changes in true oscillatory activity. Quantitative inspection of absolute spectra in our cohort indicates a steeper aperiodic slope in more severely affected patients, a hypothesis that warrants direct testing in future work. Importantly, this interpretation does not contradict prior findings but instead suggests that they may capture a distinct physiological phenomenon. Using spectral parameterization, we identified ubiquitous oscillatory activity in the alpha range around 8 Hz and 12 Hz in VC/VS recordings, as similarly reported in^19,20^. After removing the aperiodic component of spectrum, periodic spectra clearly differentiated patients based on OCD symptom severity and correlated with Y-BOCS around the individual alpha peak. A marginal difference between the groups and lack of correlation with OCD symptom severity was observed in the delta range. Together, these results support the interpretation that excessive alpha oscillations constitute a specific manifestation of dysfunctional cortico-striato-thalamo-cortical dynamics underlying OCD.

Having established the form of this physiological abnormality in OCD, the next question concerns its anatomical source. Within the spatial constraints imposed by DBS sensing, which necessarily encompasses adjacent white- and gray-matter structures, our data nonetheless indicate that periodic alpha activity is highly focal and localizes to the most anterior portion of the GPe (MNI: X = −10.12, Y = 4.07, Z = −4 mm). This localization was most robust and interpretable when using spectral parameterization, consistent with prior work in Parkinson’s disease^22^ demonstrating improved anatomical-physiological correspondence after isolating periodic components. Although OCD has traditionally been framed as a disorder resulting from hyperactivation of the direct (net excitatory) pathway^41-43^, the role of the indirect (net inhibitory) pathway remains comparatively underexplored. The anterior GPe constitutes a limbic pallidal hub that is densely interconnected with both afferent and efferent basal ganglia circuits and regulates information flow within cortico-striato-thalamo-cortical networks via the indirect pathway^44^. This region may play a critical role in exploration-exploitation behavior^45^ by regulating directed exploration^46^, interpreted as information-seeking behavior driven by uncertainty and the need to update internal models of the environment. A recent work studying schizophrenia in non-human primates^47^ has shown that GPe activity scales with directed exploration and governs the balance between information-seeking and random exploration through its interaction with prefrontal cortex. This provides a useful lens through which to interpret our findings in OCD. Whereas schizophrenia has been characterized by a jumping-to-conclusion bias^48^, reflecting premature commitment based on insufficient evidence and excessive random exploration, OCD may represent a diametrically opposed information-gathering bias, marked by excessive indecision and a pathological inefficiency of information seeking^49^. In this view, compulsive behaviors may arise not from insufficient action, but from repeated action in the absence of effective belief updating, reflecting a failure to resolve uncertainty despite extensive sampling. Importantly, converging evidence implicates pallidal dysfunction in compulsive behavior: pallidal lesions have been associated with OCD-like behavior in humans^50-53^, and selective inhibition of GPe-projecting striatal neurons reduces compulsive grooming in the Sapap3-knockout mouse model of OCD^54,55^. Taken together, we hypothesize the alpha oscillations localized to the anterior GPe may index an over-stabilized pallido–cortical control state that constrains directed exploration and impairs adaptive information gathering, thereby promoting repetitive, habitual behaviors. DBS in OCD may act by disrupting maladaptive feedback loops within the limbic GPe and re-enabling efficient information seeking and belief updating, ultimately restoring cognitive and behavioral flexibility.

In addition to the superiority of periodic spectra for symptom correlations and anatomical localization, a second consistent feature of our findings is the pronounced left-hemispheric dominance of alpha–symptom relationships. While lateralization was evident in cross-sectional analyses, it became exclusive in longitudinal within-patient analyses, where significant correlations between alpha power and Y-BOCS were observed only in the left hemisphere. Part of this asymmetry may reflect subtle differences in electrode positioning, as left-hemisphere leads were slightly closer to anterior GPe and further from BNST than right-hemisphere leads. Consequently, right-hemisphere recordings may sample a greater contribution from the BNST, a structure involved in the regulation of motivational, arousal, and emotional states^56^ and reported to exhibit reduced alpha oscillatory activity in OCD relative to major depressive disorder. A non-mutually exclusive explanation is the presence of genuine circuit-level lateralization in OCD pathophysiology, reflecting preferential vulnerability of left-hemispheric cortico-striato-thalamo-cortical networks to pathological alpha synchronization. This pattern contrasts with findings from BNST recordings in patients with major depressive disorder, where right-lateralized theta-band biomarkers have been linked to anxiety biotypes and affective outcomes^57^. Given the high prevalence of comorbid anxiety and depression in OCD, these observations argue against treating comorbidity as a nuisance variable. Instead, symptom dimensions may provide a principled basis for stratifying electrophysiological biotypes across adjacent limbic circuits and DBS targets, supporting the development of a library of psychiatric LFP biomarkers capable of guiding stimulation in a symptom-specific and circuit-sensitive manner^58^.

Our results have at least three significant clinical implications. First, active contacts selected for therapeutic stimulation exhibited higher (min-max) normalized periodic alpha power than inactive contacts at the first clinic visit. Inactive contacts, not used for chronic stimulation, showed mostly low, but also high values. While inactive contacts generally showed lower values, some displayed elevated alpha power but may not have been systematically tested or were excluded due to side effects or absence of acute behavioral responses traditionally interpreted as target engagement. These results support periodic alpha power as a relevant — though not exclusive — tool for DBS programming and suggest it may help narrow candidate contacts, substantially reducing programming burden. Second, the ability of GPe alpha activity to track longitudinal symptom fluctuations highlights its potential utility as a control signal for adaptive DBS in OCD. Importantly, this signal is recorded from the same electrodes used for stimulation, providing a practical foundation for electrophysiological-informed adaptive paradigms. However, extraction of periodic alpha power requires spectral parameterization, which currently poses challenges for real-time implementation within existing DBS platforms. Third, although the GPe is not directly targeted at our center, lead trajectories traverse its anterior limbic portion; elevated periodic alpha may therefore index an optimal stimulation 'sweet spot, similar to beta oscillations in Parkinson’s disease^59^; a hypothesis that warrants formal testing in future work. Alternatively, GPe may serve as a sensing substrate to guide stimulation delivered elsewhere along the same lead, without the need for additional electrodes. These insights may also inform non-invasive strategies aimed at modulating pallidal alpha synchronization via frontal cortico-striato-thalamo-cortical engagement.

Our findings should be interpreted in light of several limitations. First, the sample size remains relatively modest, which limits statistical power, particularly for hemisphere-specific analyses. This constraint reduced our ability to stratify results by OCD symptom dimensions, medication status, comorbid anxiety or depression, and other clinical subtypes, and precluded robust cross-validated prediction at the individual-patient level. Second, OCD symptom severity was assessed using the Yale–Brown Obsessive Compulsive Scale (Y-BOCS), which reflects overall symptom burden over the preceding two weeks and relies on retrospective self-report. Although Y-BOCS remains the clinical gold standard, it may not fully capture momentary subjective states during brief in-clinic LFP recordings or transient fluctuations in OCD severity occurring on shorter timescales. Third, LFP recordings were acquired during temporary off-stimulation periods using the BrainSense Survey mode of the Medtronic Percept^™^ system. To preserve the clinical workflow, a standardized washout period was not systematically enforced, raising the possibility that residual effects of chronic stimulation influenced neural activity. However, prior work in basal ganglia recordings suggests that stimulation-related suppression of beta oscillations typically resolves within seconds after stimulation cessation, making prolonged carryover effects unlikely^60^. Fourth, recordings were brief (~ 20 s), increasing susceptibility to physiological and movement-related artifacts and limiting the stability of spectral estimates. Although cardiac artifacts are reduced in off-stimulation Percept^™^ recordings^61^ and all segments were manually inspected, short recording durations may still bias spectral estimates, particularly in low-frequency bands that are sensitive to residual physiological activity and slow non-stationarities. Fifth, the interval between clinic visits varied substantially across and within patients, limiting methodological consistency for longitudinal analyses. Moreover, low-frequency activity in the VC/VS region is subject to circadian modulation. Although recordings were generally obtained at similar times of day across patients, circadian rhythms in OCD are heterogeneous in both amplitude and phase and may evolve during chronic stimulation^20^. Consequently, residual time-of-day effects on observed correlations cannot be fully excluded. Sixth, anatomical localization of periodic alpha activity relied on pseudo-monopolar estimation rather than true monopolar recordings. While this approach enables anatomical interpretation of bipolar LFPs, it remains an approximation and cannot definitively resolve the neural generators of the observed oscillations. Finally, the absence of a psychiatric comparison cohort limits conclusions regarding the specificity of our findings to OCD. Alpha-range oscillations may reflect partially shared dysfunctional states, such as anxiety, cognitive inflexibility, or altered arousal, that covary with OCD symptom severity. Future cross-diagnostic studies will be required to determine whether anterior GPe periodic alpha represents an OCD-specific biomarker or a trans-diagnostic electrophysiological signature.

In summary, our study identifies excessive, periodic alpha activity localized to the anterior GPe as a robust electrophysiological correlate of OCD symptom severity. These findings delineate the form of dysfunctional cortico-striato-thalamo-cortical dynamics that mediates OCD symptom expression and support a principled, electrophysiological-informed framework for DBS programming and adaptive stimulation strategies in OCD.

## Methods

### Participants

Thirteen patients diagnosed with severe, treatment-resistant obsessive-compulsive disorder (OCD) were included in this study. This study was reviewed by the Institutional Review Board (IRB) of the Massachusetts General Hospital (MGH) and considered IRB exempt as it utilized retrospective data collected during routine clinical visits according to established protocols (2024P000964). No interventions or changes in patient management were implemented as part of this study. In accordance with the standard opt-out procedure, all patients were informed and given the opportunity to object to the use of their data for research purposes. All patients had OCD for more than 6 years and failed, or were unable to tolerate, adequate trials of multiple medications that are selective serotonin reuptake inhibitors (SSRIs), clomipramine and antipsychotics, as well as expert exposure and response prevention therapy. Patient demographics are included in Table 1. Neither sex nor gender was considered in the study design, and sex/gender analyses were not performed due to the small sample size and lack of pre-existing hypotheses on sex/gender differences.

### DBS surgery

DBS leads (Model 3387 or Sensight B33015; Medtronic, Minneapolis, Minnesota, US) were placed bilaterally in the VC/VS region. Lead locations were determined using direct targeting on the preoperative MRI targeting the gray-white interface in the ventral region of the anterior limb of the internal capsule. We used the observation of positive valence response during intraoperative testing as an indicator of engagement of ventral limbic tracts that are associated with OCD and of which location in the VC/VS region that would be more promising of long-term benefit^62^. The pair of leads was connected to extensions that were tunneled to a Medtronic Percept^™^ (PC or RC) internal pulse generator (IPG).

### DBS electrode localization

DBS electrodes were localized using Lead-DBS software (http://www.lead-dbs.org)^63^. Post-operative CT and MRI scans were linearly coregistered to preoperative T1 images using Advanced Normalization Tools (ANTs, http://stnava.github.io/ANTs/)^64^. Subcortical refinement was applied (as a module in Lead-DBS) to correct for brain shift that may have occurred during surgery. Images were then normalized into ICBM 2009b Nonlinear Asymmetric (“MNI”) template space using the SyN approach implemented in ANTs, with an additional subcortical refinement step to attain a most precise subcortical alignment between patient and template space (“Effective: Low Variance” preset as implemented in Lead-DBS). Both coregistrations and normalizations were visually reviewed and refined, if needed. DBS electrodes were then localized using Lead-DBS and warped into MNI space.

### Clinical assessments

Severity of OCD symptoms was assessed during clinic visits both pre- and postoperatively using the Yale-Brown Obsessive Compulsive Scale (Y-BOCS)^65^. We considered clinical response as periods when a patient demonstrated clinically meaningful improvement in OCD symptoms defined quantitively as ≥ 35% improvement in Y-BOCS^66^ (with respect to the pre-operative score) and/or qualitatively as significant improvement of quality of life allowing return to professional/social life. During the first clinic visit, the clinical team performed an extensive monopolar review to identify the therapeutical stimulation setting that would yield optimal long-term control of OCD symptoms. The standard clinical protocol relies on the empirical evidence that VC/VS DBS exerts reversible effects on mood including the induction of mirthful smiling and laughter, as well as despair and tearfulness, which are considered as a promising sign of engagement of the VC/VS OCD circuitry. We split patients into “more severe” and “less severe” groups based on median Y-BOCS during the recording session.

### Neural recordings

Neural recordings were collected during periodic postoperative clinic visits as part of standard DBS therapy management (see Supplementary Table S1 for details). For most patients (9 out of 13; 69.2%), the first recording session (S1) occurred within 21 days of Percept device implantation, consistent with standard clinical practice, either prior to (4/13) or during (5/13) the initial clinic visits when stimulation was first programmed and activated. Four patients (OP02: 232 days, OP03: 113 days, OP07: 441 days, OP13: 504 days) experienced longer delays before the first recording session due to missing data within the expected timeframe, each attributable to specific clinical or logistical circumstances. Before any intervention in patient management, that is the adjustment of DBS settings, we used the BrainSense Survey sensing technology of the Medtronic Percept^™^ device to collect snippets of time-domain neural recordings of ~ 20 s of duration from all bipolar channel pairs at a sample rate of 250 Hz. Recordings were repeated multiple times (min:1 and max:5 across patients and sessions) if internal artefacts alerts were displayed on the Medtronic clinician programmer. These data were exported in JSON format after the clinical visits and analyzed using custom code based on the Fieldtrip^67^ toolbox implemented in MATLAB, available at (github.com/Brain-Modulation-Lab/bml).

### Signal conditioning

The data stream lost few data packets (< 0.5% across all recordings). We recovered the timestamp of missing samples by using metainformation of the received data packets (*TicksInMses* and *GlobalPacketSizes* data fields) in the JSON file^68^. Neural recordings were manually inspected to identify cardiac and motion artefacts. We were able to collect at least one recording without significant artifactual contamination in 24 hemispheres (144 bipolar channel pairs) for the first recording session S1 and up to 26 hemispheres (156 bipolar channel pairs) for other sessions and included in the next analyses. We applied a 4th -order low-pass Butterworth filter at 1 Hz to remove drifts and low-frequency components. Line noise artifacts were not removed to avoid spectral distortion but explicitly included as a narrowband component in the spectral parameterization (see *Power spectral density estimation and parameterization).* This choice was also justified by our quest of electrophysiological biomarkers of OCD symptoms in the low frequency range. No re-referencing was applied. For all analyses, we included only neural recordings for which a Y-BOCS assessment was available on the same day or, if not (< 5% of visits), within a maximum of one week.

### Power spectral density estimation: absolute and relative spectra

Power spectral densities (PSDs) were computed using the Welch^69^ method with 1-second Hanning windows (50% of overlap between contiguous windows) and a frequency resolution of 0.5 Hz. These spectra will be referred as absolute spectra. Relative power spectra in percentage units were obtained by dividing the absolute spectra by the sum in the 3-100 Hz range and multiplying by 100%. Relative power spectra were used only in supplementary analyses, while our primary focus remained on absolute and periodic power spectra (introduced in *Spectral parameterization: periodic spectra*).

### Spectral parameterization: periodic spectra

To decompose the absolute spectra in aperiodic and periodic spectral components, we used a recent elaboration of the *specparam*^24^ algorithm (formerly known as FOOOF) proposed by our group^23^ that avoids parameter unidentifiability and does not require a priori decision of whether to use the knee parameter or not. This parameterization (available at github.com/Brain-Modulation-Lab/fooof/tree/lorentzian) decomposes the log-power spectra log(P(f)) into an aperiodic component log(L(f)) and the summation of N narrow-band periodic components that are each modeled as a Gaussian:

(1) $$\log\;(P(f))=\log\;(L(f))+{\sum \;}_{n=0}^{N}a_{n}e^{-\frac{(f-{f_{c,n}}^{2}}{{2w_{n}}^{2}}}$$

where f is the frequency, $a_{n}$ is the absolute power, $f_{c,n}$ the center (or peak) frequency, and $w_{n}$ is the width of the n-th Gaussian (i.e., the standard deviation). Gaussians were used to model physiological oscillations and spectral artifacts like line noise. This approach was preferred over notch filters as the model does not adequately fit spectra with notches^25^. The aperiodic activity log log(L(f)) was parameterized as follows:

(2) $$L(f)=A\frac{{f_{k}}^{\chi }+{f_{min}}^{\chi }}{{f_{k}}^{\chi }+f^{\chi }}$$

where A is the aperiodic offset and can be interpreted as the power fitted at the minimal frequency of interest $f_{min}$, defined as the smallest positive frequency for which power can be reliably estimated based on acquisition, preprocessing and PSD estimation methods ($f_{min}=1\;\text{Hz}$ in this work). $f_{k}$ is the knee frequency at which there is a change in log-log slope of the PSD and, for $f_{k}>>f_{min}$, corresponds to the frequency at which the power decays to A∕2. The rate at which the power decreases for frequencies above $f_{k}$ is defined by the aperiodic exponent χ. The full model adequately fit cases with no knee in the absolute power spectra by converging to $f_{k}<f_{min}$ ($f_{k}<1$ or equivalently $log(f_{k})<0$). The algorithm also introduces a regularization term λ=100 in the cost function that penalizes the integral of the Gaussians over negative frequencies. We used the following *specparam* parameters: fit range: 1–65 Hz, peak width limits: 1–15 Hz, maximum number of peaks: 8, minimum peak height: 0.1 and peak threshold: 2. The periodic spectra were obtained by subtracting the aperiodic fit from the absolute spectra. The *R*^2^ was used to evaluate the goodness of fit of the model: periodic and aperiodic components were further visually inspected. We have also visualized the reconstruction absolute error to seek potential frequency specific warning in the model fit. For more details please refer to ^23^. Absolute, relative, and periodic power spectra from the first recording session S1 were contrasted frequency by frequency to evaluate hemispheric differences and group effects between less and more severe patients (see Statistical analysis).

### Alpha band and narrowband power

Alpha power was obtained from the absolute, relative, or periodic spectra by computing the average power, i.e., integral approximation using the trapezoidal method, in the canonical alpha range (7–14 Hz)^70^, which captures most of the variance of the individual peak frequencies observed in our data (Figure S4D). Note that multiple definitions of the canonical alpha range have been proposed in literature, e.g., (7–13 Hz)^71^, (8–12 Hz)^39^, (8–13 Hz)^72^, (8–14 Hz)^73^ and (9–13 Hz)^22^. We also calculated the power in a ± 2.5 *Hz* interval centered around the individual peak frequency in the alpha range (either detected by FOOOF, otherwise the local maximum), which we referred to as *peak (absolute, relative, or periodic)* ± 2.5 *Hz alpha power.*

### Analysis strategy based on alpha power

Alpha power from bipolar channel pairs was entered into two complementary analytical pipelines (Fig. 1D and Figure S3): (i) correlation with clinical outcomes (Y-BOCS scores) both at the first recording session S1 and longitudinally across sessions, and (ii) mapping onto contact-level pseudo-monopolar estimates to identify the optimal stimulating contact and for anatomical visualization at the first recording session S1. For all correlation analyses, we repeated the procedure using different hemisphere-based strategies: pooling both hemispheres, isolating the left or right hemisphere, and summarizing within each hemisphere either by averaging or by selecting the bipolar channel pair with the highest alpha power.

### Pseudo-monopolar alpha power

The anatomical and clinical interpretation of neural signals recorded from bipolar channel pairs poses a significant challenge. When a biomarker is known to predict the optimal stimulation site but is only available from bipolar recordings, localizing the source of neural activity, and thereby identifying the most effective monopolar contact, requires estimating the relative contribution of each contact to the observed signal. In the case of distant bipolar pairs (e.g., 0–2, 1–3, 0–3), it has been suggested that the optimal stimulation contact may lie either between the two contacts or at one of them^59,74^. Similarly, for adjacent bipolar pairs (e.g., 0–1, 1–2, 2–3), ambiguity remains regarding which contact underlies the recorded signal. Because monopolar recordings are not available during clinical visits, multiple methods^27,75,76^ have been developed to infer pseudo-monopolar power estimates from bipolar data. These approaches aim to map bipolar-derived power features onto individual contact levels to enable prediction of the optimal monopolar stimulation contact. In this study, we adopted a pattern-based estimation method (originally proposed by ^27^) that maps the power features of all bipolar contact pairs onto a heatmap that represents the spatial distribution of the power feature across contacts. To derive pseudo-monopolar estimates, we computed, for each contact, the maximum between two quantities: (1) the average power across all bipolar pairs that include the contact (e.g., for contact 0: pairs 0–1, 0–2, and 0–3), and (2) the power of any bipolar pairs that span across the contact but do not include it directly (e.g., for contact 1: pair 0–2). This approach yielded a contact-level estimate of power (clinic visit S1: 96 contacts), which we referred to as the pseudo-monopolar estimate of power. We applied this mapping to absolute, relative, and periodic power in the alpha band or within a narrowband centered on the individual alpha peak. Pseudo-monopolar estimates of alpha power recorded in the first recording session S1 were used as predictors to identify the optimal stimulating contact determined during the monopolar review (see Optimal contact identification) and as variables for anatomical mapping and spatial localization in MNI space (see Spatial localization of oscillations).

### Optimal contact identification

We compared pseudo-monopolar estimates of absolute and periodic alpha power between active contacts — those that produced reliable acute clinical benefit in mood, energy, and anxiety, and were therefore selected for therapeutic stimulation during the initial monopolar review (12 patients, 24 hemispheres) — and inactive contacts. Since inactive contacts were approximately three times more numerous than active ones, we controlled for sample size imbalance by performing a right-sided permutation test. Specifically, we computed the difference in the alpha power, after normalizing between 0 and 1 using the min-max normalization, between active contacts and randomly sampled subsets of inactive contacts (matched in number to the active contacts) over 10,000 iterations. Next, we applied a previously published prediction framework to assess the probability (or likelihood) of correctly identifying the optimal stimulation contact based on the ranking of contacts by electrophysiological features^77,78^. We compared two prediction strategies:

1. Chance-based prediction, simulating the clinical trial-and-error method. Assuming a DBS lead with four contacts, the probability of identifying the optimal contact increases by 0.25 with each additional contact tested, reaching 1.0 after all four have been evaluated.
2. Electrophysiology-informed prediction, where contacts were ranked according to their pseudo-monopolar alpha power (absolute and periodic). For each patient, we determined how many top-ranked contacts needed to be tested before the clinically selected (optimal) stimulation contact was reached.

We quantified prediction performance by calculating the area under the curve (AUC) of the cumulative probability of choosing the optimal stimulating contact for each method. AUC equals to 0.5 means chance-level performance while AUC equals to 1 means perfect prediction. To assess significance, we constructed a null distribution by permuting the electrophysiological rank order 10,000 times and recomputing the AUC.

Finally, to evaluate alignment between electrophysiological predictions and clinical decisions, we calculated the rank difference between the clinically selected contact (defined to have a clinical rank of 1) and its electrophysiological rank^79^. We again used a permutation test to assess whether the observed rank difference was smaller than expected by chance. For the few cases in which a double monopolar stimulation configuration was selected (8.3%: 2 hemispheres out of 24; see Supplementary Table S1), we evaluated whether either one of the two clinically optimal contacts corresponded to the first-ranked contact based on electrophysiological features.

### Spatial localization of oscillations

The spatial localization of oscillatory activity followed protocols established in previous electrophysiological DBS studies^59,74,80^, with one key modification: instead of mapping power values from bipolar channel pairs (e.g., using the midpoint between electrodes or both contact coordinates), we mapped the pseudo-monopolar estimation of the power, specifically the peak periodic alpha, at the individual contact level. To increase spatial resolution and facilitate visualization, right hemisphere contact coordinates were non-linearly flipped to the left hemisphere. For 2D visualization, we generated heatmaps by applying scattered interpolation to estimate power values between data points, followed by Gaussian smoothing (FWHM = 0.75). The resulting maps were displayed in MNI space overlaid with subcortical parcellations from the Lead-DBS toolbox: the GPe and NAcc from the OCD Response Tract Atlas^81^, and the BNST from the Human Hypothalamus Atlas^82^), all visualized against a high-resolution post-mortem MRI backdrop^83^. We then performed a series of analyses to evaluate the spatial focality of the alpha power distribution and its relationship with anatomical landmarks in the ventral capsule/ventral striatum (VC/VS) region. Specifically, we assessed (i) the correlation between alpha power and the Euclidean distance to the spatial peak (i.e., mean of the top 5th percentile) of alpha activity (spatial focality analysis), and (ii) the distance from each contact to the closest border of the GPe, NAcc, and BNST (anatomical proximity analysis). To further assess spatial focality, we implemented an additional metric based on the moment of inertia (I), a concept from physics that quantifies the spread of a mass distribution. In this context, the power value served as the weight, and the inertia I was calculated as:

(3) $$I=\frac{{\sum \;}_{i=1}^{N}w_{i}{\left\|{\bar{r}}_{i}-{\bar{r}}_{avg}\right\|}^{2}}{{\sum \;}_{i=1}^{N}w_{i}}$$

where N is the number of contacts (96), ${\bar{r}}_{i}$ is the MNI coordinates of contact i-th, $w_{i}$ is the power value at that contact, and ${\bar{r}}_{avg}$ is the weighted center of mass. The denominator ensures that I has units of mm^2^ and enables scale-invariant comparison across power definitions (e.g., absolute, and periodic alpha power). Moreover, the square root of I can be used to define the radius of gyration, representing the effective radius of an equivalent sphere that has the same moment of inertia as the observed spatial distribution. To test whether the observed spatial concentration of power was statistically significant, we performed a left-sided permutation test with 10,000 iterations. To account for interhemispheric variability and geometric constraints of lead placement, we first averaged power values within each hemisphere. We then calculated residuals by subtracting the group mean from each contact's value. These residuals were shuffled within hemispheres and added to permuted hemisphere-level means. The moment of inertia I was then recomputed from each permutation to generate a null distribution for comparison.

### Longitudinal analysis

We included neural recordings from all clinical visits in these analyses (N = 582 recordings across 12 patients). To investigate longitudinal tracking of OCD symptom severity by alpha power (both within the canonical alpha band and within a narrowband centered on the individual alpha peak), we pooled Y-BOCS scores and alpha power across all patients and sessions and performed two complementary analyses. In the first, we minimized between-patient variability by computing changes in Y-BOCS (as percentage) and alpha power (as log-ratio) relative to S1 and correlating these differences using Spearman’s rank correlation. In the second, we modeled the relationship between alpha power and symptom severity using linear mixed-effects models (LME). In the primary model, we pooled data across hemispheres and included an interaction term between hemisphere and the alpha power metric:

Y-BOCS∼Hemisphere×Alpha power metric+Stimulation amplitude+Days after DBSonset+(1∣ID)

where ID denotes patient identity and was included as a random intercept to account for repeated measurements within individuals. To further assess hemispheric effects, we repeated the analysis using a simpler model applied separately to each hemisphere:

Y-BOCS∼Alpha power metric+Stimulation amplitude+Days after DBS onset+(1∣ID)

For all models, we reported the regression coefficient (β) with its standard error (SE). Significance of the effects and interactions was assessed with post hoc F tests^84^ using the Satterthwaite method for degree-of-freedom approximation.

We also compared power spectra between the first programming session S1 and the session showing the greatest clinical improvement, defined by the largest reduction in Y-BOCS score relative to the preoperative baseline and a significant improvement in quality of life.

### Statistical analysis

We used the *RainCloudPlots* library for visualizing data distributions^85^. A Kolmogorov–Smirnov test indicated that the assumption of normality was rarely satisfied. Therefore, we employed non-parametric permutation tests (10000 permutations unless otherwise specified) throughout the manuscript whenever the definition of a null distribution was methodologically justified. This approach makes no assumptions about the underlying distribution or structure of the data. Similarly, all confidence intervals (CI) around the mean were estimated using bootstrapping with 10000 resamples and results were reported as Mean ± 95th CI, unless otherwise reported. For comparisons involving multiple frequency bins, such as condition-wise contrasts (paired test: left hemisphere vs. right hemisphere; unpaired test: less severe vs. more severe; paired test: first recording session S1 vs. best clinical improvement session) in power spectra, we applied cluster-based correction to control the family-wise error rate (*α* = 0.01), following the approach described in ^86^. Differences between proportions were tested using the Chi-squared test. Correlations were visualized using linear regression (Pearson’s fit) but calculated using Spearman’s rank correlation coefficient *R*, which is more robust to non-normality. Linear mixed-effect models were implemented using the *lme4* and *lmerTest* libraries in R. All *p*-values are reported using asterisk notation and were assessed at a significance threshold of *α* = 0.05: *p* < 0.05*, *p* < 0.01**, *p* < 0.001***.

## Supplementary Material

This is a list of supplementary files associated with this preprint. Click to download.

- VissaniOCDAlphaBiomarkerSupplementaryMaterialFinal.docx
- ExtendedDataFigures.docx

## Data Availability

The data that support the findings of this study are available from the corresponding author upon reasonable request.

## Code availability

All code used to analyze the dataset is openly available at https://github.com/Brain-Modulation-Lab/Vissani2026_alphaOCDBiomarker.
